# Occupational Noise-Induced Hearing Loss in Indian Black Tea-Processing Factories: Assessing the Prevalence and Associated Factors of an Overlooked Problem

**DOI:** 10.7759/cureus.116350

**Published:** 2026-09-16

**Authors:** Alapan Bandyopadhyay, Abhijit Mukherjee, Deyasinee Paul

**Affiliations:** 1 Community Medicine, North Bengal Medical College and Hospital, Siliguri, IND; 2 Community Medicine, Nil Ratan Sircar Medical College & Hospital, Kolkata, IND; 3 Nursing, Kalinga Institute of Nursing Sciences, Bhubaneswar, IND

**Keywords:** india, occupational noise-induced hearing loss, personal protective equipment, tea factories, workplace safety

## Abstract

Background

Occupational noise-induced hearing loss (ONIHL) is an important occupational health problem, but evidence among workers in the Indian tea-processing industry remains limited. This study aimed to estimate the prevalence of ONIHL, characterize workplace noise exposure across occupational job stations, and examine factors associated with ONIHL among workers in black tea-processing factories in Darjeeling district, West Bengal, India.

Methods

A cross-sectional study was conducted from February 2022 to April 2025 among 312 workers from six black tea-processing factories in Darjeeling district. The factories were selected by convenience sampling, followed by simple random sampling of eligible workers within participating factories. Sociodemographic and occupational information was collected using a structured questionnaire. Participants underwent otorhinolaryngological examination and pure-tone audiometry, while workplace sound levels were assessed across occupational job stations and expressed as eight-hour equivalent continuous A-weighted sound levels (Leq,8h). Multivariable logistic regression was used to examine factors associated with ONIHL after adjustment for age, sex, duration of employment, and job station.

Results

The mean age of participants was 34.1 ± 9.6 years, and 241 (77.2%) were male. ONIHL was identified in 90 of 312 workers, giving a prevalence of 28.8% (95% CI: 24.1-34.1%). The highest ONIHL prevalence occurred in the crushing-tearing-curling (CTC) room (44.8%), maintenance section (37.5%), and withering room (28.2%), which also recorded the highest Leq,8h values of 85.3, 85.1, and 80.7 dBA, respectively. After adjustment, significant associations were observed for the CTC room (adjusted OR (AOR) = 33.968; 95% CI: 8.606-134.074), withering room (AOR = 27.514; 95% CI: 5.743-131.816), maintenance section (AOR = 13.431; 95% CI: 2.478-72.798), combined sorting and packing category (AOR = 9.030; 95% CI: 1.766-46.161), and increasing age (AOR = 1.084 per year; 95% CI: 1.028-1.142). Male sex was not statistically significantly associated with ONIHL (p = 0.060). None of the workers was observed wearing hearing-protection devices during workplace assessment.

Conclusions

ONIHL was common among workers in the participating black tea-processing factories and varied substantially across occupational job stations, with the highest prevalence occurring in areas with the greatest measured workplace noise exposure. Occupational job station and increasing age were significantly associated with ONIHL, although several workstation-specific adjusted estimates were imprecise and should be interpreted cautiously. The absence of observed hearing-protection use highlights an important gap in occupational hearing-safety practices. Workstation-focused hearing-conservation measures, including noise monitoring, audiometric surveillance, engineering and administrative controls, worker education, and consistent use of hearing-protection devices, should be considered.

## Introduction

Noise-induced hearing loss (NIHL) is a permanent, bilateral sensorineural hearing impairment resulting from both brief and prolonged exposure to noise [1,2]. Before the Industrial Revolution, NIHL was caused primarily by environmental noise exposure. However, over the years, exposure to high noise levels encountered in the workplace has led to the rise of occupational NIHL (ONIHL), which has emerged as the primary form of NIHL and is recognized as the most prevalent occupational disease globally [3,4]. ONIHL affects workers across diverse industries, including construction, aviation, music, manufacturing, and agriculture [5,6].

India is one of the world’s largest tea producers, with high-yield tea-growing regions concentrated in the Eastern Himalayan foothills [7]. Tea processing involves several mechanized operations, including withering, crushing-tearing-curling (CTC), drying, sorting, packing, and equipment maintenance, which may generate substantial occupational noise. Research conducted by Çiçek and Sümer in Turkey and the authors’ previous single-factory pilot study in Darjeeling demonstrated that workers engaged in tea processing may be exposed to noise levels exceeding 85 dBA [8,9]. However, the authors’ earlier study was limited to a single factory, and evidence regarding the distribution of workplace noise exposure and ONIHL across multiple black tea-processing factories in this region remains limited.

The present study therefore extended the earlier pilot investigation to six black tea-processing factories in Darjeeling district. Specifically, the study aimed to (1) estimate the prevalence of ONIHL among black tea factory workers; (2) characterize workplace noise exposure across different occupational job stations and document observed hearing-protection practices; and (3) examine factors associated with ONIHL, particularly age, sex, duration of employment, and occupational job station.

## Materials and methods

Study design and area

An observational study with a cross-sectional design was conducted in the black tea factories of Darjeeling district, West Bengal, India, from February 2022 to April 2025.

Sample size estimation

The sample size was calculated primarily for estimation of the prevalence of ONIHL, which constituted the principal study outcome. Based on the authors’ previous pilot study conducted among black tea factory workers in Darjeeling district, the expected prevalence of ONIHL was taken as 28.3% [9]. Using Cochran’s formula for estimation of a single population proportion, as follows:

$n = \frac{Z^2p(1-p)}{d^2},$

with a 95% confidence level (Z = 1.96), an expected prevalence (p) of 0.283, a complementary proportion (q) of 0.717, and an absolute precision (d) of 0.05, the calculated sample size was 311.8, which was rounded to 312 participants. Accordingly, 52 eligible workers were recruited from each of the six participating factories to obtain the required total sample.

As the primary sample-size calculation was precision-based rather than based on a hypothesis test, an additional sensitivity power analysis was performed to assess the adequacy of the achieved sample for the comparison of ONIHL across the seven occupational job stations. At a two-sided alpha level of 0.05 and six degrees of freedom, a sample of 312 provided 80% power to detect an effect size of Cohen’s w = 0.209 and 90% power to detect an effect size of w = 0.236. The observed workstation-by-ONIHL association corresponded to an effect size of approximately w = 0.346. In addition, 90 participants with ONIHL were available for the multivariable logistic regression analysis, providing approximately 10 outcome events per parameter for the final model containing age, sex, duration of employment, and job station.

Study population and inclusion criteria

A multistage sampling methodology was utilized. In the first stage, six black tea-processing factories operating in Darjeeling district were conveniently selected as primary sampling units. Following that, from each factory, 52 employees were selected using simple random sampling from the existing pool of employees in order to reach the required sample size. Employees with less than one year of factory experience, those previously diagnosed with permanent hearing loss prior to their employment, and individuals who declined to provide written informed consent were excluded from the study. Additionally, all participants who consented underwent a thorough otorhinolaryngological examination by a specialist to rule out alternative causes of hearing loss, such as otitis media, tympanic membrane perforation, or impacted cerumen.

Audiometric assessment

Following otorhinolaryngological examination to exclude alternative causes of hearing impairment, pure-tone air-conduction audiometry was performed using a portable Arphi Electronics™ Proton SX-5 audiometer (Arphi Electronics Pvt. Ltd., Mumbai, India). The instrument conforms to applicable IEC/ANSI specifications for audiometric equipment, and calibration was maintained in accordance with the manufacturer’s recommendations. The functional operation of the audiometer and headphones was checked before testing.

Audiometric testing was undertaken in a quiet room physically separated from the active machinery and production areas of the factories. The testing environment was selected to minimize interference from ambient occupational noise, and background sound levels were assessed before audiometric examination to ensure that they were within acceptable limits for pure-tone threshold testing. Participants were instructed to remain free from significant occupational noise exposure for at least 14 hours before audiometry in order to minimize temporary threshold shift resulting from recent noise exposure. Wherever possible, examinations were therefore scheduled before commencement of the participant’s work shift or following an adequate period away from workplace noise.

Pure-tone air-conduction hearing thresholds were assessed separately for each ear across the frequencies required for identification of NIHL. Audiometric findings were interpreted in conjunction with the preceding otorhinolaryngological examination to identify participants fulfilling the study criteria for ONIHL.

Diagnosis of ONIHL

ONIHL, the primary study outcome, was defined at the participant level. A participant was classified as having ONIHL only when both ears demonstrated sensorineural hearing impairment with an average air-conduction threshold >25 dB HL across 0.5, 1, 2, and 4 kHz, together with a characteristic high-frequency noise notch. The noise notch was defined separately for each ear as a hearing threshold at 3, 4, or 6 kHz at least 10 dB poorer than the threshold at 1 or 2 kHz. Thus, both ears were required to fulfill the predefined audiometric criteria for a participant to be classified as having ONIHL. Participants with clinical or audiometric evidence suggestive of an alternative or predominantly conductive cause of hearing impairment were not classified as having ONIHL. No age-related correction of audiometric thresholds according to ISO 1999 was applied; age was instead included as a covariate in the multivariable analysis.

Air-conduction hearing thresholds were assessed at 0.5, 1, 2, 3, 4, 6, and 8 kHz. Bone-conduction testing was also performed and taken into account when determining the nature of hearing impairment, thereby allowing differentiation of sensorineural hearing loss from hearing loss with a conductive component. The audiometric ISO value was calculated from hearing thresholds at 0.5, 1, 2, and 4 kHz.

Data collection

After obtaining appropriate permissions from the study institution, a list of the six conveniently selected tea gardens was prepared, and the respective administrations of the gardens were approached. After obtaining the required permissions, lists of all employees of each of the tea factories were prepared according to the inclusion and exclusion criteria. A random number table was then utilized to select 52 employees for the present study using simple random sampling without replacement. After recruitment of the participants, written informed consent was obtained from each participant, and they were interviewed using the questionnaire to obtain sociodemographic and job-related data. Finally, the presence of NIHL was measured using a portable audiometer (Arphi Electronics™ Proton SX-5, Arphi Electronics Pvt. Ltd.) [10,11]. Use of hearing-protection devices was assessed by direct observation of participants at their respective workstations during the workplace assessment. The researchers specifically observed whether workers were wearing hearing-protection devices, including earplugs or earmuffs, while performing their usual occupational activities. Availability of hearing-protection equipment at the participating factories was also ascertained during the workplace visits. Adequate earplugs for each worker were available at each of the participating factories except one. The study flow diagram is shown in Figure 1.

**Figure 1:**
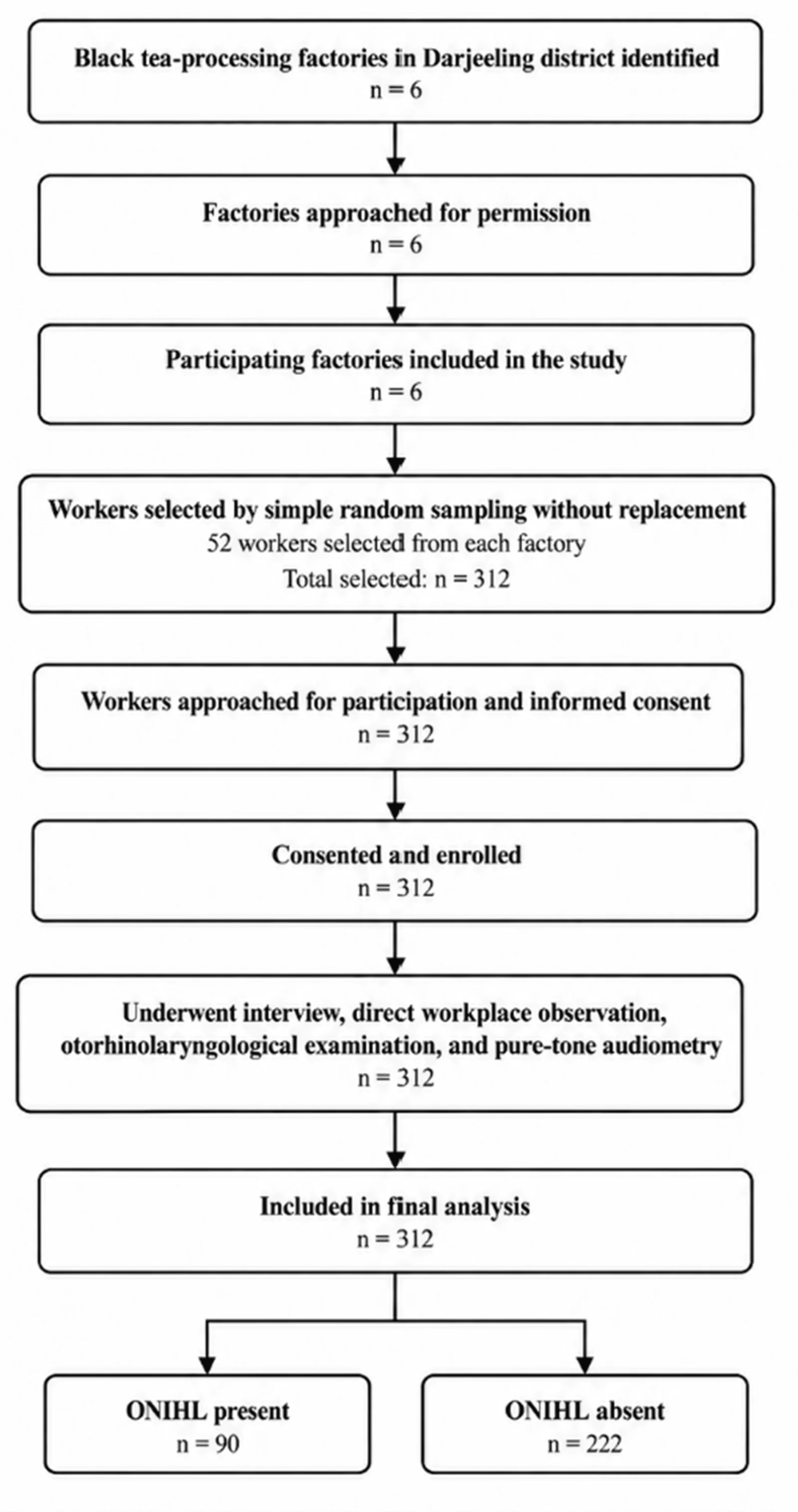
Flow diagram of the study methodology. ONIHL, occupational noise-induced hearing loss

Questionnaire 

Data were collected using a predesigned, structured, researcher-administered questionnaire based on the instrument previously used by the authors in a pilot study among black tea factory workers in Darjeeling district (Appendix A) [9]. The questionnaire was developed in accordance with the objectives of the study and included sections pertaining to sociodemographic characteristics and occupational/work-related characteristics, including current job station, duration of employment in the factory, and hearing-protection practices. Most sociodemographic information, including age, sex, educational status, and employment-related details where available, was obtained from the official records maintained by the respective factories and was supplemented or verified during the participant interview when required.

The questionnaire was pretested before commencement of the main study to assess the comprehensibility and sequence of the questions and the feasibility of administration. Based on the pretesting, necessary minor modifications were incorporated into the final questionnaire. The interviews were conducted face to face by the researchers. The original questionnaire was prepared in English. Where verbal questioning or clarification was required, questions were administered in either Hindi or Bengali, depending on the language best understood by the participant. The relevant questions were translated from English into Hindi and Bengali by native speakers of the respective languages and were independently back-translated into English by another set of speakers before administration. The original and back-translated versions were compared to ensure preservation of the intended meaning of the questionnaire items.

Information obtained from factory records and participant interviews was recorded in the study schedule. Use of hearing-protection devices at the workplace was additionally assessed by direct observation of participants at their respective workstations during routine factory activities.

Workplace noise assessment

Workplace noise assessment was undertaken using the methodology previously employed by the authors in a pilot study among black tea factory workers in Darjeeling district [9]. Sound pressure levels were measured using a portable Digital Instruments™ SL-4012 sound level meter (Lutron Electronic Enterprise Co., Ltd., Taipei City, Taiwan) conforming to IEC 61672 Class 2 specifications. Measurements were recorded in A-weighted decibels (dB(A)). The instrument was operated within its calibrated measurement range, and calibration was checked according to the manufacturer’s specifications using a 94-dB acoustic reference before workplace measurements.

Noise measurements were undertaken in the principal occupational areas of the factories, including the CTC, withering, drying, sorting, packing, maintenance, and administrative sections. Initially, the locations generating the highest sound pressure levels within each work area were identified at approximately worker ear level. Additional measurements were subsequently obtained at worker stations at incremental distances from these locations to characterize spatial variation in noise exposure. Therefore, rather than using a predetermined fixed number of sampling points in each work area, measurement locations corresponded to the actual working stations and relevant noise zones occupied by employees during routine factory operation. Worker movements and the time spent at different workstations during an eight-hour shift were determined by direct observation.

For each identified workstation, sound pressure levels were measured throughout an eight-hour working shift at 15-minute intervals. At each observation, five sound-pressure-level readings were obtained over five seconds at one-second intervals, and their arithmetic mean was recorded as the representative level for that observation. Measurements were performed while routine machinery and production activities were in operation. The full-shift measurement procedure was repeated across the three factory work shifts on three nonconsecutive working days to account for within-day and between-day variation, and the measurements were averaged.

The workstation-specific observations generated over the working shift were entered into the web-based Noise Exposure Calculator of NoiseMeters to calculate the eight-hour equivalent continuous A-weighted sound level (Leq,8h). For workers occupying more than one workstation during their shift, workstation-specific exposure levels were combined with the duration spent at each location to estimate daily Leq,8h exposure. The ranges presented for individual work areas represent the observed variation in sound pressure levels across measurement locations and sessions, whereas Leq,8h represents the corresponding eight-hour equivalent exposure.

Data analysis

Participant characteristics were summarized using frequencies and percentages for categorical variables and means with standard deviations for continuous variables. The prevalence of ONIHL was calculated as the proportion of participants fulfilling the audiometric criteria for ONIHL among all participants examined. The overall prevalence and workstation-specific prevalence estimates were reported with 95% CIs calculated using the Wilson score method for binomial proportions. Pearson’s chi-square test was used to assess the unadjusted association between job station and the presence of ONIHL. Multivariable binary logistic regression was subsequently performed with ONIHL status as the dependent variable and age, sex, duration of employment in the factory, and job station as independent variables. Administrative office staff and female sex were used as the respective reference categories. Associations were expressed as adjusted ORs (AORs) with 95% CIs.

Leq,8h was not entered as a separate independent variable in the multivariable model because each occupational job station was assigned a single representative Leq,8h value. Consequently, Leq,8h was deterministically defined by job station and was completely aliased with the corresponding job-station indicator variables. Simultaneous inclusion of both terms would therefore result in exact linear dependence and a rank-deficient model. Job station was retained as the occupational exposure variable, while Leq,8h was reported descriptively to characterize the noise environment of each work area. Variance inflation factors were not used for this decision because the dependence between these two variables was structural rather than an empirical degree of collinearity. A two-sided p-value of <0.05 was considered statistically significant.

Ethical considerations

The study was conducted after obtaining ethical clearance from the Institutional Ethics Committee of the North Bengal Medical College and Hospital and necessary permissions from the respective authorities. Written informed consent was obtained from each participant before the interview, and privacy, confidentiality, and anonymity were maintained for all study participants.

Participants with audiometric findings suggestive of ONIHL were individually informed of their screening results and counseled regarding occupational noise exposure and the importance of hearing-protection measures. They were advised to seek further assessment by an otorhinolaryngologist or audiologist for confirmatory evaluation, determination of the extent of hearing impairment, and appropriate clinical management. Workplace-level findings, including the distribution of noise exposure and the identification of higher-noise work areas, were communicated to the respective factory administrations in aggregate form to facilitate appropriate occupational hearing-conservation measures. Individual-level audiometric findings were not disclosed to factory management without the participant’s consent, and participant confidentiality was maintained throughout the process.

The study was reported in accordance with the Strengthening the Reporting of Observational Studies in Epidemiology (STROBE) statement for cross-sectional studies.

## Results

A total of 312 black tea factory workers were included in the study. The mean age of the participants was 34.1 ± 9.6 years, with the largest proportion belonging to the 30-39-year age group (116, 37.2%), followed by those aged <30 years (114, 36.5%). Most participants were male (241, 77.2%), while 71 (22.8%) were female. Regarding educational status, 104 (33.3%) had completed secondary education, 66 (21.2%) had completed primary education, 43 (13.8%) had completed higher secondary education, and 27 (8.7%) were illiterate. Most participants belonged to the lower socioeconomic classes, with 118 (37.8%) classified as Class IV and 71 (22.8%) as Class V according to the modified B.G. Prasad socioeconomic classification. The CTC room constituted the largest occupational group, comprising 134 (42.9%) workers, followed by the withering room and administrative office, with 39 (12.5%) participants each. Regarding duration of employment, 131 (41.9%) participants had worked in the factory for 5-10 years, 112 (35.9%) for >10 years, and 69 (22.0%) for <5 years (Table 1).

**Table 1:** Clinicosocial parameters of the participants (n = 312). Mean ± SD for duration of job (years): 10.7 ± 8.8 years CTC, crushing-tearing-curling

Parameters	Frequency	Percentage
Age (years)		
<30	114	36.5
30-39	116	37.2
40-49	44	14.1
50 and above	38	12.2
Sex		
Female	71	22.8
Male	241	77.2
Education		
Illiterate	27	8.7
Non-formal	34	10.9
Primary	66	21.2
Middle	23	7.4
Secondary	104	33.3
Higher secondary	43	13.8
Graduate	15	4.8
Socioeconomic status		
Class I (Rs 9098 and above)	0	0
Class II (Rs 4549-9097)	38	12.2
Class III (Rs 2729-4548)	85	27.2
Class IV (Rs 1364-2728)	118	37.8
Class V (<1364)	71	22.8
Job station		
CTC room	134	42.9
Withering room	39	12.5
Drying room	29	9.3
Sorting room	26	8.3
Packing room	29	9.3
Maintenance staff	16	5.1
Administrative office staff	39	12.5
Duration of job (years)		
<5 years	69	22
5-10 years	131	41.9
>10 years	112	35.9

During direct workplace observation, none of the 312 participants was observed wearing a hearing-protection device (0/312; 0.0%), including earplugs or earmuffs, while performing their usual occupational activities. Hearing-protection equipment was available in five of the six participating factories, although observed worker use was absent.

Additional audiometric characteristics are presented in Appendices B and C. Frequency-specific threshold assessment demonstrated relative preservation of hearing at the lower frequencies, with greater threshold elevation across 3-6 kHz and the greatest mean impairment at 4 kHz. The frequency-specific impairment was most pronounced among maintenance and CTC workers. Among participants classified as having ONIHL, all had bilateral involvement in accordance with the predefined diagnostic criteria. Most cases were characterized by mild hearing impairment, and the predominant audiometric notch occurred at 4 kHz. The distribution of sex across occupational job stations is shown in Appendix D. Sex was unevenly distributed across work areas, with male workers predominating in the CTC, sorting, maintenance, and administrative sections, while females predominated in the packing room (Appendices B-D).

It was observed that noise exposure differed substantially across the occupational job stations. The CTC room recorded a noise exposure range of 82-86 dBA with an Leq,8h of 85.3 dBA, while maintenance workers experienced the widest noise range of 55-93 dBA with an Leq,8h of 85.1 dBA. The withering room had noise levels ranging from 70-84 dBA with an Leq,8h of 80.7 dBA. Comparatively lower Leq,8h values were recorded in the drying room (72.8 dBA; range 66-74 dBA), sorting room (66.7 dBA; range 62-74 dBA), packing room (66.5 dBA; range 60-74 dBA), and administrative office (60.6 dBA; range 50-66 dBA). The prevalence of ONIHL was highest among workers in the CTC room, where 60 of 134 workers (44.8%) had ONIHL. This was followed by maintenance staff, with 6 of 16 workers (37.5%), and workers in the withering room, with 11 of 39 (28.2%). The corresponding prevalence was 13.8% (4/29) among packing-room workers, 10.3% (3/29) among drying-room workers, 10.2% (4/39) among administrative office staff, and 7.7% (2/26) among sorting-room workers. The distribution of ONIHL differed significantly according to job station (χ² = 37.434, p < 0.001). Because the expected ONIHL count for the maintenance category was below five, a Monte Carlo exact significance test was additionally performed, which confirmed the association (Monte Carlo p < 0.001). ONIHL was detected in 90 of the 312 participants, giving an overall prevalence of 28.8% (95% CI: 24.1-34.1%) (Table 2).

**Table 2:** ONIHL prevalence among the participants (n = 312). * Statistically significant. One expected cell count was <5 (minimum expected count = 4.62); therefore, statistical significance was additionally verified using a Monte Carlo exact test (p < 0.001). CTC, crushing-tearing-curling; ONIHL, occupational noise-induced hearing loss

Job station	Noise level exposure, dBA			ONIHL present		ONIHL absent		ONIHL prevalence, n/N (%)	95% CI	Chi-square value	p-value
	Range	Leq, 8h, dBA	Median (IQR)	Frequency	Percentage	Frequency	Percentage				
CTC room	82-86	85.3	85.2 (84.0-86.9)	60	66.7	74	33.3	60/134 (44.8)	36.6-53.2	37.434	<0.001*
Withering room	70-84	80.7	79.8 (77.8-81.6)	11	12.2	28	12.6	11/39 (28.2)	16.5-43.8		
Drying room	66-74	72.8	73.8 (72.2-74.2)	3	3.3	26	11.7	3/29 (10.3)	3.6-26.4		
Sorting room	62-74	66.7	66.6 (66.4-67.1)	2	2.2	24	10.8	2/26 (7.7)	2.1-24.1		
Packing room	60-74	66.5	66.8 (66.1-67.2)	4	4.4	25	11.3	4/29 (13.8)	5.5-30.6		
Maintenance staff	55-93	85.1	83.1 (81.8-88.9)	6	6.7	10	4.5	6/16 (37.5)	18.5-61.4		
Administrative office staff	50-66	60.6	62.6 (62.1-63.4)	4	4.4	35	15.8	4/39 (10.2)	4.1-23.6		
Total	-	-	-	90	100	222	100	90/312 (28.8)	24.1-34.1		

Multivariable binary logistic regression was performed to examine factors independently associated with ONIHL after adjustment for age, sex, duration of employment, and job station. Because of the small numbers of participants and ONIHL events within some occupational categories, the sorting and packing rooms were combined for the regression analysis. These two work areas had nearly identical equivalent eight-hour noise exposure levels (Leq,8h 66.7 and 66.5 dBA, respectively), overlapping measured noise ranges, and relatively low numbers of ONIHL events, making their combination appropriate for reducing sparse cells while retaining a meaningful occupational exposure category. Administrative office staff and female participants were used as the reference categories. Compared with administrative office staff, higher adjusted odds of ONIHL were observed among workers in the CTC room (AOR = 33.968; 95% CI: 8.606-134.074; p < 0.001), withering room (AOR = 27.514; 95% CI: 5.743-131.816; p < 0.001), combined sorting and packing rooms (AOR = 9.030; 95% CI: 1.766-46.161; p = 0.008), and maintenance section (AOR = 13.431; 95% CI: 2.478-72.798; p = 0.003). The association for the drying room was not statistically significant (AOR = 2.924; 95% CI: 0.509-16.784; p = 0.229). Increasing age remained independently associated with ONIHL (AOR = 1.084 per one-year increase; 95% CI: 1.028-1.142; p = 0.003). Male sex showed higher adjusted odds of ONIHL, although the association did not reach statistical significance (AOR = 2.132; 95% CI: 0.969-4.689; p = 0.060). Duration of employment was also not independently associated with ONIHL (AOR = 1.031 per year; 95% CI: 0.975-1.089; p = 0.281). The model demonstrated reasonably good discrimination (c-statistic = 0.803), although the Hosmer-Lemeshow test was significant (χ² = 23.09, df = 8; p = 0.003), indicating imperfect calibration and supporting cautious interpretation of the adjusted estimates (Table 3).

**Table 3:** Multivariable logistic regression analysis of factors associated with ONIHL among the participants (n = 312). ^*^ Statistically significant, adjusted for age, sex, years of work in the factory, and job station. AOR, adjusted OR; CTC, crushing-tearing-curling; ONIHL, occupational noise-induced hearing loss

Parameter	AOR	95% CI		p-value
		Lower bound	Upper bound	
Job station				
CTC room	33.968	8.606	134.074	<0.001*
Withering room	27.514	5.743	131.816	<0.001*
Drying room	2.924	0.509	16.784	0.229
Sorting and packing rooms	9.03	1.766	46.161	0.008*
Maintenance staff	13.431	2.478	72.798	0.003*
Administrative office staff	1	-	-	-
Sex				
Male	2.132	0.969	4.689	0.06
Female	1	-	-	-
Age (years)	1.084	1.028	1.142	0.003*
Years of work in factory (years)	1.031	0.975	1.089	0.281

In an exploratory sensitivity analysis, cumulative occupational noise exposure was represented by an index calculated as the representative workstation Leq,8h multiplied by the participant’s exact duration of employment. The cumulative exposure index was higher among participants with ONIHL (1188.1 ± 757.4 dBA-years) than among those without ONIHL (673.7 ± 577.1 dBA-years) and remained significantly associated with ONIHL after adjustment for age and sex. Each 100 dBA-year increase in the cumulative exposure index was associated with 10.4% higher adjusted odds of ONIHL (AOR = 1.104; 95% CI: 1.038-1.174; p = 0.002). This sensitivity analysis supported a cumulative exposure-response association despite the absence of an independent association with duration of employment alone (Appendix E).

## Discussion

The present study demonstrates a substantial burden of ONIHL among workers employed in black tea-processing factories in Darjeeling district. Among 312 workers, 90 had ONIHL, corresponding to an overall prevalence of 28.8%. This finding is consistent with the authors’ earlier pilot study and supports the presence of an occupational hearing hazard in tea-processing environments [9]. Tea factories in this region are generally located away from major urban and industrial noise sources, making factory machinery an important source of occupational noise exposure [9,12]. Rastogi et al. also highlighted ONIHL as an important but inadequately addressed workplace problem in India [13].

Noise exposure varied considerably across job stations. The highest Leq,8h was recorded in the CTC room at 85.3 dBA, followed closely by maintenance work at 85.1 dBA, while the withering room recorded 80.7 dBA. Lower levels were observed in drying, sorting, packing, and administrative areas. The prevalence of ONIHL followed a similar occupational pattern, being highest among CTC workers at 44.8%, followed by maintenance staff at 37.5% and withering workers at 28.2%. The association between job station and ONIHL was statistically significant (χ² = 37.434, p < 0.001), indicating marked variation in hearing-loss burden across work areas.

Multivariable analysis showed that job station remained strongly associated with ONIHL after adjustment for age, sex, and duration of employment. Compared with administrative office staff, statistically significant associations were observed for workers in the CTC room, withering room, maintenance section, and the combined sorting and packing category, whereas the association for the drying room was not statistically significant. Although consolidation of the sorting and packing categories reduced sparse cells, relatively small numbers of ONIHL events remained in several occupational categories. Consequently, the large AORs and wide CIs indicate statistical imprecision and possible residual sparse-data bias. The workstation-specific adjusted estimates should therefore be interpreted primarily as evidence of heterogeneity in association rather than as precise estimates of effect magnitude. Greater emphasis was accordingly placed on the descriptive correspondence between measured workplace noise levels and ONIHL prevalence across occupational job stations.

Increasing age remained independently associated with ONIHL. Duration of employment, when modeled independently as a continuous per-year variable, was not significantly associated with ONIHL after adjustment for age, sex, and job station. This finding should not necessarily be interpreted as evidence for the absence of a cumulative occupational effect. Because the study included only workers who remained employed at the time of the cross-sectional survey, a healthy worker survivor effect is possible. Workers who developed clinically important hearing impairment, experienced difficulty tolerating occupational noise, or were transferred or left employment because of health concerns would not have been represented among longer-serving current workers. Such selective retention of comparatively healthier workers could attenuate an association between employment duration and ONIHL. Consistent with this possibility, the exploratory sensitivity analysis incorporating both duration and workstation noise exposure demonstrated a significant association between the cumulative exposure index and ONIHL, with a 10.4% increase in adjusted odds for every 100 dBA-years of the index. This finding provides additional evidence that both the intensity and duration of occupational noise exposure may be relevant when considered jointly. However, because the index was derived from workstation-level Leq,8h values rather than longitudinal personal dosimetry and because dBA is a logarithmic measure, it should be regarded as a relative cumulative-exposure proxy rather than a direct quantitative measure of lifetime acoustic energy exposure. Male workers had higher adjusted odds of ONIHL than female workers, but this association did not reach conventional statistical significance in the revised model. These findings suggest that age and occupational work area may contribute to differences in ONIHL occurrence; however, residual differences in actual machinery exposure, proximity to noise sources, movement between workstations, previous occupational exposure, and cumulative lifetime noise exposure could not be fully accounted for in the present analysis.

An important occupational-safety finding was the absence of observed hearing-protection use among the study participants. During direct observation at their respective workstations, none of the 312 workers was observed wearing hearing-protection devices, including earplugs and earmuffs. This finding did not appear to result from a complete lack of availability, as earplugs were available at all participating factories except one. The principal gap therefore appeared to involve uptake and adherence to available hearing-protection measures rather than provision alone. This is concerning in areas such as the CTC and maintenance sections, where Leq,8h approached or exceeded 85 dBA. Recent National Institute for Occupational Safety and Health guidelines emphasized the role of personal protective equipment education in reducing occupational risks, supporting the need for structured worker education alongside provision of protective devices [14]. Similarly, Khoza-Shangase highlighted gaps and barriers in the prevention of ONIHL and the need for effective prevention strategies [15]. These observations support hearing-conservation programs involving sound monitoring, regular audiometry, engineering controls, preventive machine maintenance, administrative exposure reduction, and consistent use of hearing protection.

The socioeconomic profile of the participants may also have occupational-health implications. Most workers belonged to lower socioeconomic classes, and few had higher educational attainment. Adei et al. reported an association of demographic and socioeconomic factors with occupational injuries and diseases among informal-sector workers, supporting consideration of social determinants when designing workplace safety interventions [16].

Limitations

The study has several limitations. Its cross-sectional design precludes causal inference and may be affected by a healthy worker survivor effect, as workers with greater hearing impairment may have transferred to quieter duties or left employment before the survey. The six factories were conveniently selected from a single district, and workers were clustered within factories without explicit adjustment for intrafactory correlation. Equal numbers of workers were sampled from each factory irrespective of workforce size and without sampling weights; consequently, workers from smaller factories may have been overrepresented, and those from larger factories underrepresented, and the overall prevalence should be interpreted as an unweighted estimate for the participating sample. The 38-month sequential data-collection period may also have introduced temporal heterogeneity due to changes in machinery, maintenance, production intensity, or work organization.

Several established determinants of sensorineural hearing loss, including previous and nonoccupational noise exposure, ototoxic medication use, diabetes, hypertension, smoking, alcohol consumption, and personal listening-device use, were not assessed, leaving the possibility of residual confounding. Small numbers of participants and ONIHL events in some work areas resulted in wide CIs and unstable workstation-specific estimates, while imperfect model calibration further warrants cautious interpretation. Audiometry was performed using a portable audiometer in a quiet room rather than a dedicated sound-treated booth; residual ambient noise may therefore have affected hearing thresholds, particularly at lower frequencies. Finally, repeated area-based noise measurements rather than personal dosimetry may not fully represent individual noise dose or capture worker mobility, variations in distance and orientation relative to machinery, intermittent task-specific exposures, short-duration peaks, or within-shift changes in machine operation.

## Conclusions

ONIHL was common among workers employed in the participating black tea-processing factories, with an overall prevalence of 28.8%. ONIHL prevalence varied substantially across occupational job stations and was highest among workers in the CTC room, maintenance section, and withering areas, which also recorded the highest workplace noise levels. After adjustment for age, sex, and duration of employment, occupational job station remained significantly associated with ONIHL, although the magnitude of several workstation-specific estimates was imprecise because of wide CIs. Increasing age was also significantly associated with ONIHL, whereas the association with male sex did not reach statistical significance. Given the cross-sectional design, these findings should be interpreted as associations rather than evidence of causal effects. The observed differences across work areas highlight the importance of workstation-focused occupational hearing-conservation measures. The absence of observed hearing-protection use despite the availability of earplugs in most participating factories identifies an important gap in hearing-safety practices. Regular workplace noise monitoring, periodic audiometric screening, engineering and administrative noise-control measures, preventive machine maintenance, worker education, and appropriate use of hearing-protection devices should be considered as components of occupational hearing-conservation programs in tea-processing factories.

## Appendices

Appendix A

Study questionnaire

Identification details

Item information

Participant ID __________________

Factory code __________________

Date of interview ____ / ____ / ______

Interviewer code/name __________________

Language used for interview: ☐ English ☐ Bengali ☐ Hindi

Section A. Sociodemographic characteristics

A1. Age

Age at last birthday: ______ years

For analysis:

☐ <30 years

☐ 30-39 years

☐ 40-49 years

☐ ≥50 years

A2. Sex

☐ Male

☐ Female

A3. Educational status

Highest level of education completed:

☐ Illiterate

☐ Nonformal education

☐ Primary education

☐ Middle school

☐ Secondary education

☐ Higher secondary education

☐ Graduate or above

A4. Socioeconomic status

Monthly per-capita family income: ₹ __________________

Modified B.G. Prasad socioeconomic class:

☐ Class I - ₹9,098 and above

☐ Class II - ₹4,549-9,097

☐ Class III - ₹2,729-4,548

☐ Class IV - ₹1,364-2,728

☐ Class V - <₹1,364

Section B. Occupational characteristics

B1. Current job station

At which section/job station of the tea factory are you currently employed?

☐ Crushing-tearing-curling (CTC) room

☐ Withering room

☐ Drying room

☐ Sorting room

☐ Packing room

☐ Maintenance section

☐ Administrative office

B2. Duration of employment

For how long have you been working in this tea factory?

______ years ______ months

For analysis:

☐ <5 years

☐ 5-10 years

☐ >10 years

B3. Current working duration

Usual duration of one work shift: ______ hours

Usual work shift:

☐ Shift 1

☐ Shift 2

☐ Shift 3

☐ Other: __________________

B4. Workstation movement

During a usual work shift, do you work at more than one workstation?

☐ No

☐ Yes

If yes, record the workstations and approximate duration spent at each:

Workstation approximate duration during an eight-hour shift

__________________ ______ hours/minutes

__________________ ______ hours/minutes

__________________ ______ hours/minutes

__________________ ______ hours/minutes

Section C. Hearing-protection practices

C1. Availability of hearing-protection device

Is any hearing-protection device provided/available to you at your workplace?

☐ Yes

☐ No

☐ Do not know

If yes, type available:

☐ Earplug

☐ Earmuff

☐ Both earplug and earmuff

☐ Other: __________________

C2. Use of hearing protection

Do you use a hearing-protection device while working in the factory?

☐ Always

☐ Often

☐ Sometimes

☐ Rarely

☐ Never

C3. Type usually used

If a hearing-protection device is used, specify:

☐ Earplug

☐ Earmuff

☐ Both

☐ Other: __________________

☐ Not applicable

C4. Direct observational verification (to be completed by researcher)

Was the participant observed wearing a hearing-protection device while performing his/her

usual work activity?

☐ Yes

☐ No

If yes:

☐ Earplug

☐ Earmuff

☐ Other: __________________

Was hearing-protection equipment available at the participant's factory/workstation?

☐ Yes

☐ No

☐ Could not be verified

Section D. Verification from factory records

Variable interview response factory record verified/final entry

Age ______ ______ ______

Sex ______ ______ ______

Educational status ______ ______ ______

Current job station ______ ______ ______

Duration of employment ______ ______ ______

Section E. Researcher-recorded clinical and audiometric information

E1. Otorhinolaryngological examination

Clinical examination performed:

☐ Yes ☐ No

Evidence of another cause of hearing impairment:

☐ No

☐ Otitis media

☐ Tympanic membrane perforation

☐ Impacted cerumen

☐ Other: __________________

Eligible for audiometric assessment/classification of ONIHL:

☐ Yes ☐ No

E2. Pure-tone audiometry

Audiometer: Arphi Electronics™ Proton SX-5

Right ear

Frequency hearing threshold, dB HL

0.5 kHz ______

1 kHz ______

2 kHz ______

3 kHz ______

Frequency hearing threshold, dB HL

4 kHz ______

6 kHz ______

8 kHz ______

Left ear

Frequency hearing threshold, dB HL

0.5 kHz ______

1 kHz ______

2 kHz ______

3 kHz ______

4 kHz ______

6 kHz ______

8 kHz ______

Bone-conduction assessment performed:

☐ Yes ☐ No

E3. Audiometric ISO value

Right ear ISO value: ______ dB HL

Left ear ISO value: ______ dB HL

E4. Presence of high-frequency noise notch

Right ear

☐ Present

☐ Absent

Predominant frequency:

☐ 3 kHz

☐ 4 kHz

☐ 6 kHz

Left Ear

☐ Present

☐ Absent

Predominant frequency:

☐ 3 kHz

☐ 4 kHz

☐ 6 kHz

Section F. Final classification

ONIHL

☐ Yes

☐ No

Severity of hearing impairment

☐ Mild: 26-40 dB HL

☐ Moderate: 41-60 dB HL

☐ Severe: 61-80 dB HL

☐ Profound: >80 dB HL

☐ No ONIHL

Laterality:

☐ Bilateral

☐ Unilateral

☐ Not applicable

Predominant audiometric notch:

☐ 3 kHz

☐ 4 kHz

☐ 6 kHz

☐ Not applicable

Investigator’s certification

I certify that the above information was obtained from the participant, verified from available factory records where applicable, and supplemented by direct workplace observation and clinical/audiometric assessment according to the study protocol.

Name/signature of investigator: __________________________

Date: ____ / ____ / ______

Participant ID: __________________

Appendix B

**Table 4:** Sensitivity analysis of cumulative occupational noise exposure and ONIH. AOR, adjusted OR; ONIH, occupational noise-induced hearing loss * Statistically significant

Parameter	AOR	95% CI	p-value
Cumulative exposure index, per 100 dBA-years	1.104	1.038-1.174	0.002*
Age, per year	1.004	0.963-1.046	0.862
Male sex	1.689	0.814-3.505	0.159
Female sex	1	Reference	-

Appendix C

**Table 5:** Frequency-specific hearing thresholds according to occupational job station. Hearing thresholds are expressed in dB HL. The frequency-specific profile demonstrates relative preservation of lower-frequency thresholds with greater impairment at 3-6 kHz, with the greatest mean threshold shift at 4 kHz, consistent with the characteristic audiometric configuration of ONIHL. CTC, crushing-tearing-curling; ONIHL, occupational noise-induced hearing loss

Occupational job station	n	0.5 kHz	1 kHz	2 kHz	3 kHz	4 kHz	6 kHz	8 kHz
CTC room	134	18.9 ± 11.5	19.5 ± 12.0	21.9 ± 11.6	30.5 ± 15.6	36.5 ± 16.3	34.4 ± 15.9	28.6 ± 12.4
Withering room	39	14.2 ± 7.1	15.1 ± 7.7	17.6 ± 7.0	24.1 ± 11.1	29.0 ± 12.7	26.3 ± 10.7	23.1 ± 8.3
Drying room	29	13.5 ± 6.4	13.1 ± 7.1	15.8 ± 6.8	21.3 ± 8.0	25.7 ± 10.0	24.3 ± 10.6	21.7 ± 7.3
Sorting room	26	11.1 ± 3.2	11.8 ± 4.3	13.1 ± 3.9	17.5 ± 6.1	21.6 ± 7.7	19.7 ± 6.3	17.8 ± 5.1
Packing room	29	11.9 ± 4.9	12.9 ± 5.8	14.4 ± 5.1	19.0 ± 8.2	23.2 ± 9.4	21.7 ± 8.9	19.5 ± 6.3
Maintenance staff	16	25.0 ± 20.1	25.6 ± 21.3	29.8 ± 21.3	37.8 ± 25.3	44.4 ± 27.8	41.2 ± 26.0	36.2 ± 21.4
Administrative office staff	39	13.3 ± 4.6	13.5 ± 4.7	16.6 ± 4.4	21.0 ± 7.1	26.8 ± 8.5	24.4 ± 7.2	22.6 ± 4.8
Overall	312	16.1 ± 10.3	16.6 ± 10.8	19.1 ± 10.6	25.9 ± 14.3	31.3 ± 15.6	29.1 ± 14.9	25.2 ± 11.5

Appendix D

**Table 6:** Audiometric characteristics of participants with ONIHL. ONIHL, occupational noise-induced hearing loss * Statistically significant

Audiometric characteristic	n	%
Severity of hearing impairment*		
Mild (26-40 dB HL)	63	70
Moderate (41-60 dB HL)	22	24.4
Severe (61-80 dB HL)	5	5.6
Profound (>80 dB HL)	0	0
Laterality		
Bilateral	90	100
Unilateral	0	0
Predominant frequency of audiometric notch		
3 kHz	9	10
4 kHz	65	72.2
6 kHz	16	17.8
Total ONIHL	90	100

Appendix E

**Table 7:** Distribution of sex according to occupational job station. CTC, crushing-tearing-curling

Occupational job station	Male, n (%)	Female, n (%)	Total, n
CTC room	113 (84.3)	21 (15.7)	134
Withering room	25 (64.1)	14 (35.9)	39
Drying room	19 (65.5)	10 (34.5)	29
Sorting room	23 (88.5)	3 (11.5)	26
Packing room	9 (31.0)	20 (69.0)	29
Maintenance staff	13 (81.3)	3 (18.8)	16
Administrative office staff	39 (100.0)	0 (0.0)	39
Total	241 (77.2)	71 (22.8)	312
